# CRABP2 regulates invasion and metastasis of breast cancer through hippo pathway dependent on ER status

**DOI:** 10.1186/s13046-019-1345-2

**Published:** 2019-08-16

**Authors:** Xuefei Feng, Miao Zhang, Bo Wang, Can Zhou, Yudong Mu, Juan Li, Xiaoxu Liu, Yaochun Wang, Zhangjun Song, Peijun Liu

**Affiliations:** 1 Center for Translational Medicine, the First Affiliated Hospital of Xi’an Jiaotong University, 277 Yanta Western Rd, Xi’an, 710061 Shaanxi Province China; 2 Key Laboratory for Tumor Precision Medicine of Shaanxi Province, the First Affiliated Hospital of Xi’an Jiaotong University, 277 Yanta Western Rd, Xi’an, 710061 Shaanxi Province China; 3 Department of Breast Surgery, the first Affiliated Hospital of Xi’an Jiaotong University, 277 Yanta Western Rd, Xi’an, 710061 Shaanxi Province China; 4 Department of Clinical LaboratoryTumor Hospital of Shaanxi Province, Affiliated to the Medical College of Xi’an Jiaotong University, 277 Yanta Western Rd, Xi’an, 710061 Shaanxi Province China; 5 Department of Breast Disease Center, Tumor Hospital of Shaanxi Province, Affiliated to the Medical College of Xi’an Jiaotong University, 309 Yanta Western Rd, Xi’an, 710061 Shaanxi Province China

**Keywords:** CRABP2, Invasion and metastasis, Breast cancer

## Abstract

**Background:**

Triple Negative Breast cancer (TNBC) is incurable cancer with higher rates of relapse and shorter overall survival compared with other subtypes of breast cancer. Cellular retinoic acid binding protein 2 (CRABP2) belongs to fatty acid binding protein (FABP) family which binds with all-trans retinoic acid (RA). Previous studies from the database have reported the patients with high expression of CRABP2 showed different prognosis in ER^+^ and ER^−^ breast cancer. However, its biological role and exact mechanism in breast cancer remain unknown. This aim of this study was to explore how CRABP2 regulated invasion and metastasis based on the estrogen receptor-α (herein called ER) status in breast cancer.

**Methods:**

Immunohistochemical staining method was used to analyze the expression of CRABP2 in human breast cancer tissues. Lentivirus vector-based shRNA technique was used to test the functional relevance of CRABP2 knockdown in breast tumors. Tail vein injection model was used to examine the lung metastasis. Co-immunoprecipitation, Western blotting, immunofluorescence, and quantitative reverse transcription polymerase chain reaction (RT-qPCR) were conducted to investigate the underlying mechanism that influenced the ER to the regulation of CRABP2 to Lats1.

**Results:**

We observed that knockdown of CRABP2 promotes EMT, invasion and metastasis of ER^+^ breast cancer cells in vitro and in vivo, whereas overexpression of CRABP2 yields the reverse results. In ER^+^ mammary cancer cells, the interaction of CRABP2 and Lats1 suppress the ubiquitination of Lats1 to activate Hippo pathway to inhibit the invasion and metastasis of ER^+^ mammary cancer. However, in ER^−^ mammary cancer cells, the interaction of CRABP2 and Lats1 promote the ubiquitination of Lats1 to inactivate Hippo pathway to promote the invasion and metastasis of ER^−^ mammary cancer.

**Conclusions:**

Our findings indicate that CRABP2 can suppress invasion and metastasis of ER^+^ breast cancer and promote invasion and metastasis of ER^−^ breast cancer by regulating the stability of Lats1 in vitro and in vivo, and it provides new ideas for breast cancer therapy.

**Electronic supplementary material:**

The online version of this article (10.1186/s13046-019-1345-2) contains supplementary material, which is available to authorized users.

## Background

Breast cancer is the leading cause of death from cancer among women [1]. Patients with Triple Negative Breast cancer (TNBC) are currently the subgroup with the worst outcome [2, 3]. At present treatments options for TNBC has been limited. In order to seek out new treatments for TNBC, we found differential protein CRABP2 in MCF-10A, MCF-7, and MDA-MB-231 cells. Therefore, this study focuses on the biological function and expression of CRABP2 in human tumors.

Cellular Retinoic Acid Binding Protein 2 (CRABP2) is a small intracellular protein, which belongs to the intracellular lipid-binding proteins family [4]. The expression of CRABP2 is restricted in the skin, ovary, breast, and testis of adults [5–8]. CRABP2 transports RA to the retinoic acid receptor (RAR) in the nucleus and regulates cell proliferation, apoptosis, invasion, and metastasis [9, 10]. Previous reports state that CRABP2 can function as a transcriptional coactivator [11]. Also, it is found that CRABP2 can affect the biological behavior independently of either RA or its receptor [12]. Abnormal expression of CRABP2 is associated with malignant cancers in the human being. CRABP2 promotes pancreatic cancer cell invasion and metastasis by stabilizing the interleukin 8 expressions [13]. CRABP2 is extensively connected in the development of neuroblastoma, Wilms tumor, head and neck squamous-cell carcinoma (HNSCC), and non-small cell lung cancer (NSCLC). Reduction of CRABP2 level will suppress the movement of cancer cells [14–17]. A decrease in the number of receptors on the surface of (down-regulation) CRABP2 expression will restrain the cell proliferation in vitro and in vivo. Also, this will induce apoptosis as well as block cell metastasis in esophageal squamous cell carcinoma (ESCC) [18]. CRABP2 is candidate subtype-specific biomarker for ovarian cancers [19]. Besides, CRABP2 is strongly associated with breast cancer. CRABP2 prevents the growth of breast cancer cells by two different mechanisms [20]. Previous studies report that estrogen receptor α (ERα, also referred to as ER) regulates the transcription of CRABP2 in some way [21, 22]. On the other hand, there is evidence that favor and opposes the role of CRABP2 in breast cancer. One study stated that a high level of CRABP2 mRNA relates to a better prognosis of patients with breast cancer [7]. Conversely, another report indicates that high level of CRABP2 leads to the poor prognosis of patients with breast cancer [23]. Comparing these data leads to confusion and identifying the function of CRABP2 in breast cancer becomes difficult. Therefore, the role of CRABP2 in regulating breast cancer invasion and metastasis and the reason that high expression of CRABP2 showed different prognosis in ER^+^ and ER^−^ breast cancer remains unclear.

The Hippo pathway controls organ development through the regulation of apoptosis and cell proliferation. Recently, a growing body of researches has validated that the Hippo pathway is closely related to breast cancer cells proliferation, survival, invasion, and metastasis [24]. Large tumor suppressor 1 (Lats1) is a core component of the Hippo pathway and is vastly related to low lymph node metastasis of breast cancers [25]. However, the role of the Hippo pathway associated with breast cancer remains unclear without much research.

ER is a nuclear sex steroid receptor (SSR) expressed in about 75% of breast cancers [26]. Several findings report that ER promotes the growth of breast cancer cells [27–29]. However, researches are indicating that the loss of ER leads to the occurrence of epithelial-mesenchymal transition (EMT) and tumor metastasis [30–32]. Also, it is known that ER was stabilized in the absence of Lats1, and ER was targeted for ubiquitination in the presence of Lats1 [32]. However, the association of ER and Lats1 with breast cancer metastasis remains unclear.

Therefore, this study predominantly focuses on exploring the role of CRABP2 in different types of breast cancer in vitro and in vivo. Our results show that CRABP2 controls metastasis and invasion of breast cancer through the Hippo pathway. In addition, CRABP2 intervenes ubiquitination of Lats1 in breast cancer cells based on ER status.

## Materials and methods

### Cell lines and cell culture condition

MDA-MB-231 and BT549 cells were obtained from Shanghai Institute of Biochemistry and Cell Biology, Chinese Academy of Sciences in Shanghai respectively. T47D cells were obtained from Beijing union medical college hospital cell resource sharing platform. MCF7 cells were a gift from Dr. Jianmin Zhang. MDA-MB-468 cells were obtained from Academy of Military Sciences in Beijing. MCF7 and MDA-MB-231 cells were cultured in DMEM medium with 10% FBS and 1% Penicillin–Streptomycin. T47D cells were cultured in 1640 medium 10% FBS, 10 μg/ml insulin (Sigma) and 1% Penicillin–Streptomycin. BT549 cells were cultured in 1640 medium with 10% FBS, 1 μg/ml insulin (Sigma) and 1% Penicillin–Streptomycin. MDA-MB-468 cells were cultured in 1640 medium 10% FBS and 1% Penicillin–Streptomycin. Medium, FBS and Penicillin–Streptomycin were obtained from Hyclone. The total cells were incubated in a certain environment (5% CO_2_, 37 °C).

### Wound healing assays

Various cells were seeded in cell-culture dishes. The cells were scratched with pipette tip (10 μl) and then washed twice using PBS to remove the float when the cells nearly achieved 100% confluence. Then the cells were incubated in a certain environment (serum-free). Wound healing was quantified by measuring the percent wound closure of cells.

### Cell migration and invasion assays

2 × 10^5^ cells in 200 μl of specific medium (serum-free) were seeded in the chamber above (8-μm pore non-coated polycarbonate transwell inserts). Serum-containing medium was put in the chamber below. After some time, cells were fixed in methanol. After 10 min, cells were stained for 15 min with 0.5% crystal violet. After gently removing the cells on the up side of the top chamber, migrated and invaded cells were photographed and counted.

### RNA isolation, real-time RT-PCR

The extract of total RNA was obtained from the E.Z.N.A.® Total RNA kit I (Omega Bio-Tek, Inc., Norcross, GA, USA) and converted to cDNA with the PrimeScript™ RT Master Mix [Takara Biotechnology (Dalian) Co., Ltd., Liaoning, China]. Real-time polymerase chain reaction (real-time PCR) was conducted with the SYBR-Green I kit [Takara Biotechnology (Dalian) Co., Ltd.]. Primers are listed in Additional file 2: Table S2.

### Immunohistochemistry

The patients samples in breast cancer and adjacent non-cancerous tissue patients were from the First Affiliated Hospital of Xi’an Jiaotong University. And our study was permitted by the Ethics Committee on Human Research of the First Affiliated Hospital of Xi’an Jiaotong University.

The 4-μm-thick paraffin sections, which were roasted (60 °C, 6 h), deparaffinized in xylene and rehydrated in gradient concentration ethanol. The antigen repair of slides was performed in sodium citrate buffer (PH = 9.0) in microwave oven (100w 3 min, 50w 13 min). 3% hydrogen peroxide to deactivate endogenous peroxidase for some time (10 min) was must to be done after natural cooling of sections. After the slides were cleaned three times by phosphate buffered saline (PBS), followed by blocking with 5% BSA (Bull Serum Albumin) at 37 °C for 30 min and incubation with the primary antibody in a certain environment (4 °C, overnight). The second day, the slides were cleaned three times (3 min each time) by PBS and incubated in homologous secondary antibody (one hour, room temperature). 3-times PBS washing, diaminobenzidine staining, hematoxylin staining, dehydrating in graded ethanol and transparent in xylene to handle the slides.

The scoring method was as follows: 10 random fields of view were selected for each tissue section, and semi-quantitative scoring was performed for tissue staining in each field of view. Positive cell rate integral method: 0, no positive cells or < 10% positive cells; 1, positive cells accounted for 10%~ 25%; 2, positive cells accounted for 25%~ 50%; 3, positive cells accounted for 50%~ 75%; 4, the proportion of positive cells > 75%. Dyeing strength integral method: 0, cells without staining; 1, color is light yellow; 2, color is brown-yellow; 3, color is tan. The total score is the product of positive cell rate and staining intensity: 0 as negative; 1~4 as weak positive; 5~8 as positive; 9~12 as strongly positive.

### Preparation of cell extracts, and Western blotting

Proteins extract were obtained from RIPA lysis buffer [EDTA (1 mM), NaCl (150 mM), Tris-HCl (50 mM, pH = 7.4), sodium deoxycholate (1%), Triton X-100(1%) and SDS (0.1%)] containing phosphatase inhibitors and protease inhibitor (Roche, NJ, USA) and then were centrifuged 20 min (12,000 rpm, 4 °C). The protein lysates were electrophoresed using SDS-PAGE and transferred to polyvinyl difluoride (PVDF) (Bio-Rad, CA, USA). Membranes were blocked with 5% nonfat milk for two hours and incubated with the primary antibody in a certain environment (4 °C, overnight). The second day, membranes were cleaned three times (10 min each time) by TBST and incubated in HRP-conjugated secondary antibody (Proteintech) (one hour, room temperature). The membranes were photographed by the chemiluminescence reagent (Millipore). LaminA/C and GADPH act as controls. Nuclear and Cytoplasmic Extraction Reagents (Pioneer Biotechnology, Xi’an, China) was used for cell fractionation assays. Mst2, p-Lats1^T1079^, Lats1, p-YAP ^S127^, YAP, E-cadherin and ZO-1 antibodies were obtained from CST (Cell Signaling Technology, USA.) The LaminA/C, GADPH, Vimentin primary antibodies were obtained from Proteintech, China. The CRABP1, CRABP2 and ER primary antibodies were obtained from Abcam, UK. The Flag primary antibodies were obtained from Sigma.

### Co-immunoprecipitation

Proteins extract were obtained from immunoprecipitation lysis buffer [NaCl (150 mM), NP-40 (0.5%), Tris-HCl (pH = 8.0), glycerol (20 mM, 20%)] containing phosphatase inhibitors and protease inhibitor (Roche, NJ, USA) and then were centrifuged 20 min (12,000 rpm, 4 °C). Thermo Scientific Pierce Co-IP kit (Thermo Fisher Scientific) was used for Co-IP experiments. These obtained proteins were tested by SDS-PAGE (10%) and immunoblotted with Lats1 (Cell Signaling Technology) and CRABP2 (Proteintech) antibodies as Western blotting analysis.

### Immunofluorescence

Cells were fixed (4% paraformaldehyde), permeabilized (0.2% Triton X-100), and blocked (5% BSA). Cells were incubated with specific primary antibodies and then an Alexa Fluor 488 secondary antibody (Invitrogen) (1:200). E-cadherin (1:200), ZO-1(1:50), YAP (1:100) primary antibodies were obtained from CST. CRABP2 (1:50) primary antibody was obtained from Abnova. Vimentin (1:50) primary antibody was obtained from Proteintech. DNA staining was performed using DAPI. Microscopic analyses were conducted with a confocal laser scanning microscope (Leica TCS SP5).

### Luciferase reporter assay

Reporter gene transfection and luciferase activity assay were performed as follow: cells on a 24 well plate were co-transfected with the firefly luciferase reporter (50 ng) along with the Renilla luciferase reporter (Promega) (20 ng) for 24 h using Lipofectamine 2000 (Thermo Fisher Scientific, America) according to the protocols provided by manufacturers. The reporter plasmid of TEAD was purchased from Addgene (Addgene, America). The luciferase activity was measured in cellular extracts using a dual luciferase reported gene assay kit (Promega). The relative activity of the reporter gene was calculated by dividing the signals from firefly luciferase reporter by the signals obtained from Renilla luciferase reporter.

### In vivo tumor experiment

Four-week-old SCID-Beige female mice were from the Experimental Animal Center of the Medical College of Xi’an Jiaotong University and all animal experiments were done by protocols approved by the Institutional Animal Care and Use Committee of the First Affiliated Hospital of Xi’an Jiaotong University. The methods were consistent with the approved guidelines. In this experiment, 1 × 10^7^ MCF7 -sh-NC, MCF7- sh-CRABP2, 2 × 10^6^ MDA-MB-231-Con and MDA-MB-231 Flag-CRABP2 cells suspended in 200 ml of PBS were injected into SCID female mice via the tail vein (*n* = 6). At 5 weeks after injection, lungs were removed, photographed. The lungs were surgically fixed (4% paraformaldehyde) for hematoxylin-eosin for staining and immunohistochemistry. Relative number of metastatic lung nodules of lung tissues of mice was counted.

### Plasmid transfection, RNA interference and lentiviral infection

Human CRABP2 small hairpin RNA (shRNA) and OE-CRABP2 lentivirus were obtained from GeneChem (Shanghai, China). MDA-MB-468, T47D, MCF7 and BT549 cells infected with lentivirus and taken MOI = 20 as the standard. MDA-MB-231 cells infected with lentivirus and taken MOI = 10 as the standard. BT549 and MDA-MB-231 cells infected with OE-ER lentivirus and taken MOI = 10 as the standard. All cells were infected with lentivirus for sh-NC expression (sh-NC) or control lentivirus (Con). Seventy-six hours after infection, the cells were treated with puromycin for three weeks to get stable cells. Stably transfected MDA-MB-468, T47D, MCF7 and BT549 cells were selected by 0.75 mg/mL puromycin (Cayman Chemical, Ann Arbor, USA) in culture. Stably transfected MDA-MB-231 cells were selected by 2 mg/mL puromycin in culture.

The pcDNA3.1-CRABP2 plasmid and ER plasmid was constructed by us. The Flag-Lats1 plasmid and Flag-ER was obtained from Gene Chem. The Lats1 and ER siRNA molecules were obtained from Gene Pharma, Shanghai, China.

siRNA and Plasmid were transfected into cells using Lipofectamine 2000 (Invitrogen, Carlsbad, CA). All sequences were listed in Additional file 2: Table S3.

### Ubiquitination assay in vivo

Plasmids expressing ubiquitin-HA, Flag-Lats1 and so on were infected to cells transiently by Lipofectamine 2000. An additional 24 h MG-132 (5 μmol/L, Sigma) treating for cells was necessary after 24 h. NP-40 lysis buffer was used to treat cells. Co-IP was conducted with mouse anti-Flag M2 monoclonal antibody. Western blotting was used to detect ubiquitinated-Lats1.

### Statistical analysis

All experiments were repeated at least three times in vitro, and all data were analyzed with the GraphPad Prism 5 (GraphPad Software, Inc., La Jolla, CA, USA). These results are expressed as the mean ± S.D. Comparisons between two groups were performed using Student^,^s *t*-test. Two-way ANOVA followed by Dunnett’s multiple comparisons test was used for multiple comparisons. All statistical tests were two-sided. *P* < 0.05 was considered as statistically significant.

## Results

### The different expression and survival of patients of CRABP2 in ER^+^ and ER^−^ breast cancer

**Fig. 1:**
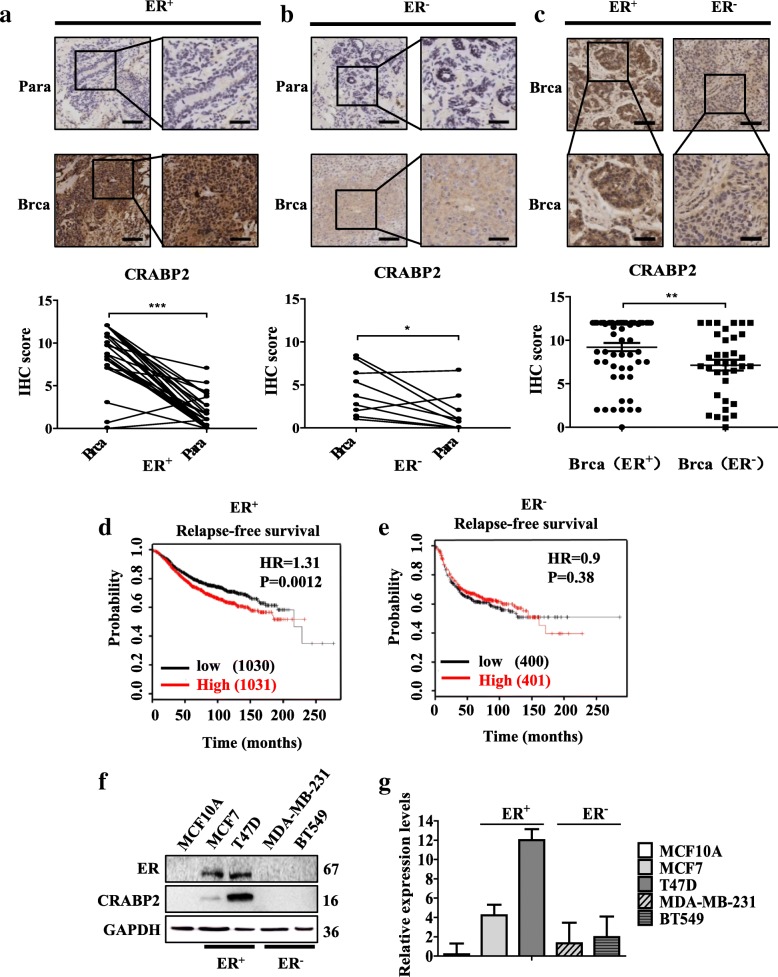
The different expression and survival of patients of CRABP2 in ER^+^ and ER^−^ breast cancer. **a-b** Representative pictures of CRABP2 in human ER^+^ (**a**) and ER^−^ (**b**) breast cancer tissues and the neighboring non-cancerous tissues with immunohistochemical staining. Scalebar, 145 μm, 73 μm. *P*-values were calculated by the Student’s *t*-test. **c** Compiled results of CRABP2 immunohistochemical staining performed on human ER^+^ and ER^−^ BRCA samples. Scalebar, 145 μm, 73 μm. *P*-values were calculated by the Student’s *t*-test. **d-e** Kaplan Meier survival curve of CRABP2 expression respectively in ER^+^ (**d**) and ER^−^ (**e**) mammary cancer. *P*-values were calculated by the log-rank test. **f-g** Expression of CRABP2 protein (**f**) and mRNA (**g**) was examined in 5 kinds of cells by Western blotting and RT-qPCR. Data are the mean ± S.D. of three independent experiments performed in triplicate. *, *P* < 0.05; **, *P* < 0.01; ***, *P* < 0.001

To investigate the function of CRABP2 in mammary cancer progression, immunohistochemical staining method was used to analyze the expression of CRABP2 in 40 pairs of human breast cancer tissues and 57 nonpaired human breast cancer tissues. The results showed that CRABP2 expression was higher in cancer tissues than in the matched surrounding tissues of ER^+^ breast cancer. (Fig. 1a). CRABP2 showed extremely faint staining in ER^−^ breast cancer and in the matched surrounding tissues, but CRABP2 expression was higher in cancer tissues than in the matched surrounding tissues of ER^−^ breast cancer (Fig. 1b). Above all, we found that CRABP2 was more highly expressed in ER^+^ than in ER^−^ breast cancer tissues (Fig. 1c). These results implied that CRABP2 was correlated with ER. Next, from the clinicopathologic status of mammary cancer, we investigated the expression levels of CRABP2 and their association with the patients. High CRABP2 protein levels indeed correlated with ER status (*p* = 0.0016) by Pearson χ^2^ test analysis (Additional file 2: Table S1). Previous studied reports that ER regulated the expression of CRABP2 and CRABP2 had opposite correlation with prognosis of patients with breast cancer [7, 21–23]. Our results showed that ER indeed regulated the expression of CRABP2 (Additional file 1: Figure S1a-b). To better understand the relationship between CRABP2 and prognosis of patients with breast cancer, we analyzed it in ER^+^ and ER^−^ breast cancer respectively by Kaplan-Meier analysis. Our results showed that the low CRABP2 expression level in ER^+^ mammary cancer tissues significantly related to a reduction in patient relapse-free survival (RFS) (Fig. 1d). At the same time, the high CRABP2 expression level in ER^−^ mammary cancer tissues related to a reduction in patient RFS by Kaplan–Meier analysis (Fig. 1e). These results indicated that CRABP2 may have different functions in ER^+^ and ER^−^ mammary cancer. Also, protein and mRNA of CRABP2 was detected and found that they were significantly higher in ER^+^ mammary cancer cells (T47D and MCF7) than in ER^−^ mammary cancer cells (BT549 and MDA-MB-231) (Fig. 1f-g). CRABP2 was barely expressed in immortalized cells (MCF10A) (Fig. 1f-g). These results confirm that the expression of CRABP2 is related to the development of breast cancer, which is related to ER.

### Knockdown of CRABP2 promotes EMT, metastasis and invasion of ER^+^ breast cancer cells in vitro and in vivo

**Fig. 2:**
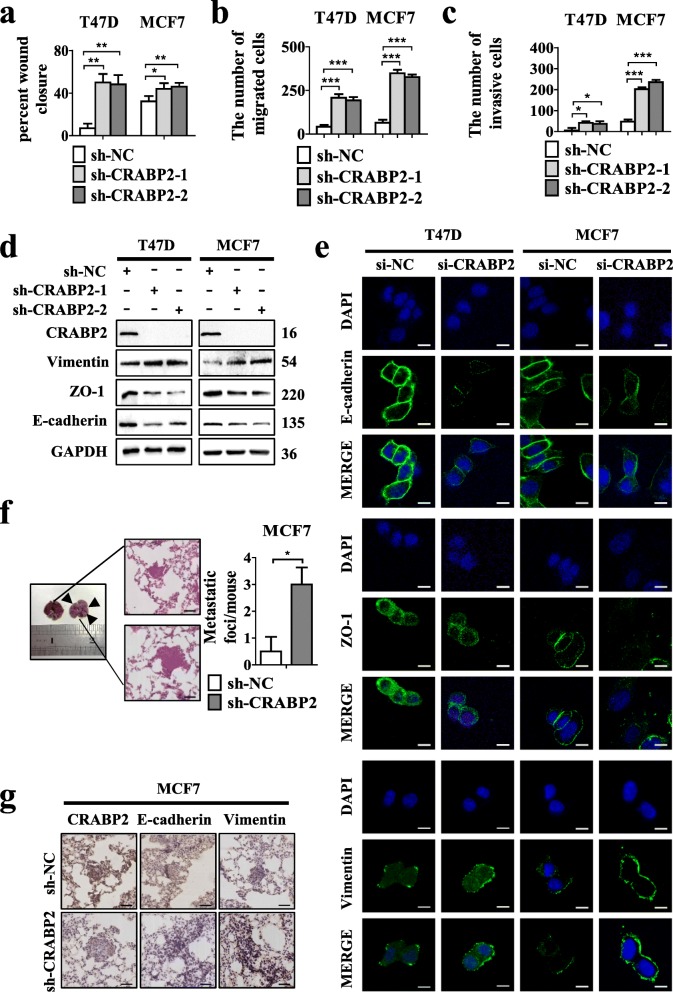
Knockdown of CRABP2 promotes EMT, metastasis and invasion of ER^+^ breast cancer cells in vitro and in vivo. (**a**) Wound-healing, (**b**) migration and (**c**) invasion experiments were conducted in wild-type cells (sh-NC), T47D and MCF7 cells with stable knockdown of CRABP2 (sh-CRABP2–1, sh-CRABP2–2). The percent of wound closure and migratory and invasive cells numbers were counted. *P*-values were calculated by the Student’s *t*-test. **d-e** Analysis of E-cadherin, ZO-1, and Vimentin expression in T47D and MCF7 cells expressing CRABP2 shRNA (sh-CRABP2–1, sh-CRABP2–2) or CRABP2 siRNA (si-CRABP2)and shRNA (sh-NC) or siRNA(si-NC) by Western blotting (**d**) and immunofluorescence (**e**). Scalebar, 10 μm. **f** Representative images, HE staining and relative number of metastatic lung nodules of lung tissues of mice. (*n* = 6 per group). **g** Immunohistochemical staining of CRABP2, E-cadherin, Vimentin in sh-NC group and sh-CRABP2 group. Scalebar, 145 μm. Data are the mean ± S.D. of three independent experiments performed in triplicate. *, *P* < 0.05; **, *P* < 0.01; ***, *P* < 0.001

Lentivirus vector-based shRNA technique was used to test the functional relevance of CRABP2 knockdown in breast tumors. We stably depleted CRABP2 in ER^+^ mammary cancer cells using this technique. Later, we used Western blotting and quantitative reverse transcription polymerase chain reaction (RT-qPCR) to detect the knockdown efficiency in T47D and MCF7 cells (Additional file 1: Figure S1c-d). We found that deficient CRABP2 promoted ER^+^ breast cancer cells migration and invasion. In monolayer wound healing and transwell assays, CRABP2-depleted T47D and MCF7 cells migrated and invaded faster than the control cells (Fig. 2a-c, Additional file 1: Figure S2a-c).

EMT-TFs play vital roles in tumor invasion and metastasis. Our results showed that knockdown of CRABP2 cells decreased expression of E-cadherin, ZO-1 and increased the expression of Vimentin (Fig. 2d-e). Ectopic expression of CRABP2 in MCF7 cells can up-regulate the protein expression of E-cadherin, ZO-1 and down-regulate the protein expression of Vimentin. (Additional file 1: Figure S2d). Therefore, these results suggest that knockdown of CRABP2 promotes ER^+^ breast cancer cells EMT, invasion, and metastasis in vitro.

Then investigations were carried out using xenografts to find out whether knockdown of CRABP2 accelerated migration and invasion in vivo*.* In a tail vein injection model, sh-NC-MCF7 and sh-CRABP2-MCF7 cells were chosen for examining the lung metastasis. This mimic the process of loss of CRABP2 during metastasis in ER^+^ patients. Pictures of impassive lungs were taken after 5 weeks. CRABP2-depleted group had notably increased metastatic nodules (Fig. 2f). Further, immunohistochemical staining results confirmed that E-cadherin expression decreased and Vimentin expression increased in the CRABP2-depleted group (Fig. 2g). Therefore, knockdown of CRABP2 promotes EMT, invasion and metastasis of ER^+^ breast cancer cells in vitro and in vivo.

### Overexpression of CRABP2 promotes EMT, metastasis and invasion of ER^−^ breast cancer cells in vitro and in vivo

**Fig. 3:**
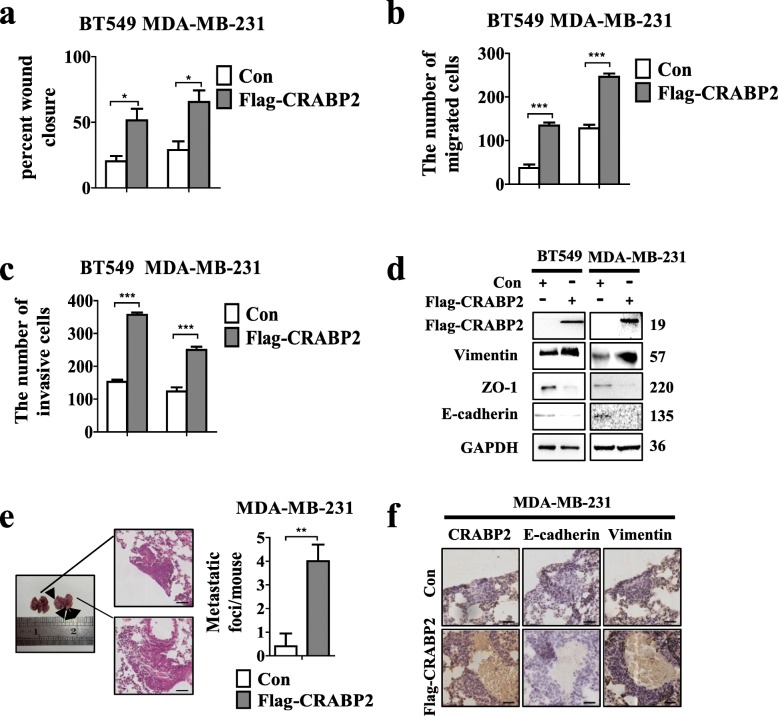
Overexpression of CRABP2 promotes EMT, metastasis and invasion of ER^−^ breast cancer cells in vitro and in vivo. (**a**) Wound-healing, (**b**) migration and (**c**) invasion experiments were conducted in wild-type cells (Con) and BT549 and MDA-MB-231 cells with stable overexpress of CRABP2 (Flag-CRABP2). The percent of wound closure and migratory and invasive cells numbers were counted. *P*-values were calculated by the Student’s *t*-test. **d** Analysis of the E-cadherin, ZO-1, and Vimentin expression in BT549 and MDA-MB-231 cells stably expressing CRABP2 (Flag-CRABP2) and vector (con) by Western blotting. **e** Representative images, HE staining and relative number of metastatic lung nodules of lung tissues of mice. (n = 6 per group). **f** Immunohistochemical staining of CRABP2, E-cadherin, Vimentin in Con group and Flag-CRABP2 group. Scalebar, 145 μm. Data are the mean ± S.D. of three independent experiments performed in triplicate. *, *P* < 0.05; **, *P* < 0.01; ***, *P* < 0.001

We continued our studies to further study the relevance of CRABP2 overexpression in ER^−^ mammary cancer by firmly overexpressing CRABP2 in ER^−^ breast cancer cells. Western blotting and RT-qPCR methods were used to verify the overexpression efficiency in BT549 and MDA-MB-231 cells (Additional file 1: Figure S1e-f). Exogenous CRABP2 expression in BT549 and MDA-MB-231 cells promoted ER^−^ breast cancer cells invasion and metastasis by monolayer wound healing and transwell assays (Fig. 3a-c, Additional file 1: Figure S3a-c). Overexpression of CRABP2 cells increased Vimentin expression and decreased E-cadherin, ZO-1 expressions (Fig. 3d). Our results in vivo found that the number of metastatic nodules was largely increased in the CRABP2-overexpressed group (Fig. 3e). Further, immunohistochemical staining results confirmed that E-cadherin expression decreased and Vimentin expression increased in the CRABP2-overexpressed group (Fig. 3f). Therefore, the ectopic expression of CRABP2 promotes EMT, invasion, and metastasis of ER^−^ breast cancer cells in vitro and in vivo. Also, it is confirmed that CRABP2 may have different effect on invasion and metastasis in ER^+^ and ER^−^ mammary cancer cells.

### CRABP2 suppresses EMT, metastasis and invasion of ER^+^ breast cancer cells by activating Hippo pathway

**Fig. 4:**
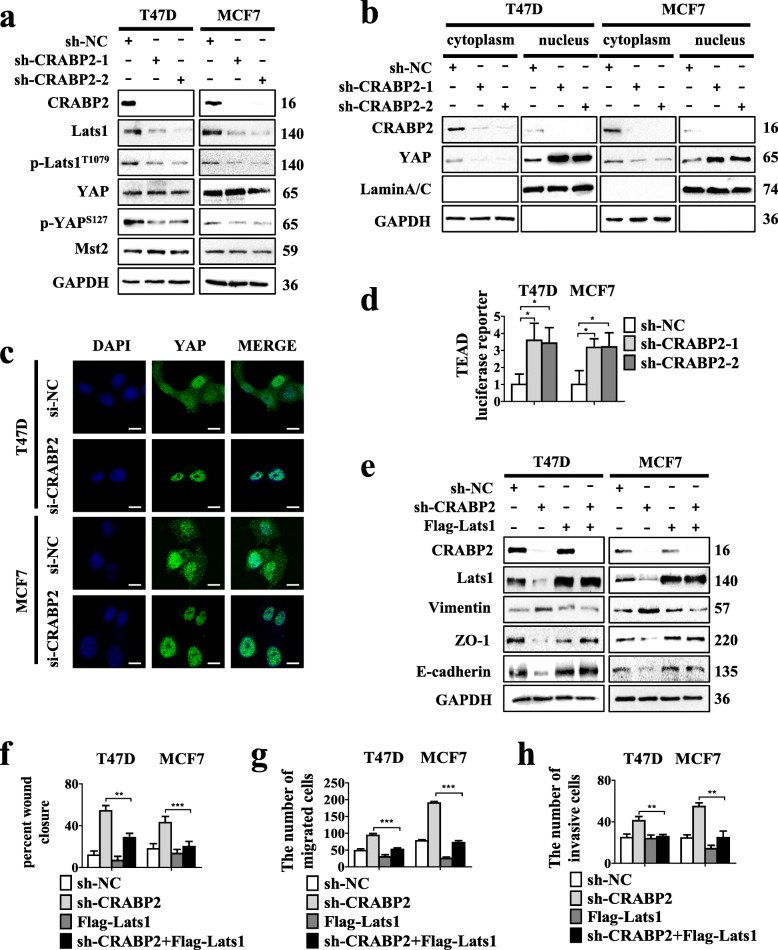
CRABP2 suppresses EMT, metastasis and invasion of ER^+^ breast cancer cells by activating Hippo pathway. **a.** Analysis of the YAP, p-YAP^s127^, Lats1, p-Lats1^T1079^ and Mst2 expression in T47D and MCF7 cells stably expressing CRABP2 shRNA (sh-CRABP2–1, sh-CRABP2–2) and shRNA (sh-NC) by Western blotting. **b.** The express of YAP was analyzed in T47D and MCF7 cells stably expressing CRABP2 shRNA (sh-CRABP1, sh-CRABP2–2) and shRNA (sh-NC) by subcellular fractionation. Western blotting of GAPDH and LaminA/C were used as controls for cytoplasmic and nuclear fractional purity, respectively. **c** The expression of YAP was analyzed in T47D and MCF7 cells expressing CRABP2 siRNA (si-CRABP2) and siRNA (si-NC) by immunofluorescence. Scalebar, 10 μm. **d** TEAD luciferase activity was analyzed in T47D and MCF7 cells stably expressing CRABP2 shRNA (sh-CRABP2–1, sh-CRABP2–2) and shRNA (sh-NC). **e** Analysis of CRABP2, Lats1, E-cadherin, ZO-1 and Vimentin expression in T47D and MCF7 cells stably expressing CRABP2 shRNA (sh-CRABP2–1, sh-CRABP2–2) and shRNA (sh-NC), with or without Lats1 (Flag-Lats1) by Western blotting. (**f**) Wound-healing, (**g**) migration and (**h**) invasion experiments were conducted in T47D and MCF7 cells stably expressing CRABP2 shRNA (sh-CRABP2–1, sh-CRABP2–2) and shRNA (sh-NC), with or without Lats1 (Flag-Lats1). The percent of wound closure and migratory and invasive cells numbers were counted. *P*-values were calculated by the Student’s *t*-test. Data are the mean ± S.D. of three independent experiments performed in triplicate. *, *P* < 0.05; **, *P* < 0.01; ***, *P* < 0.001

Now, we know that ER drives the transcription of RARα and subsequently RARα drives the transcription of CRABP2 in ER^+^ mammary cancer cells [21]. In the absence of Lats1, Lats1 stabilized ER and the Hippo effector YAP [32]. Thus, we must now delineate the association between CRABP2 and Lats1. Knockdown of Lats1 in ER^+^ breast cancer cells could not modify the mRNA and protein expression of CRABP2 (Additional file 1: Figure S4a-b). However, the protein expression of p-Lats1^T1079^, Lats1, p-YAP^S127^ in the Hippo pathway were reduced, maintaining the expression levels of YAP and Mst2 when knocking down CRABP2 in ER^+^ breast cancer cells (Fig. 4a). Likewise, the protein expression of p-Lats1^T1079^, Lats1, p-YAP^S127^ increased maintaining the expression levels of YAP and Mst2 unaltered when overexpressing CRABP2 in MCF7 cells (Additional file 1: Figure S2d). Phosphorylation of YAP promotes the cytoplasmic localization and phosphorylation of YAP in ser127 leads to cytoplasmic retention. The cellular fractionation and immunofluorescence experiments showed that knocking down CRABP2 expression in T47D and MCF7 cells reduced the YAP expression in the cytoplasm and increased the YAP expression in nucleus respectively when compared with controls (Fig. 4b-c). YAP facilitates regulation of downstream target gene expression by directly binding to the transcription factor TEADs [33, 34]. Then we checked the effects of CRABP2 depletion on TEAD luciferase activity in ER^+^ breast cancer cells. We observed that CRABP2 depletion increased TEAD luciferase activity. (Fig. 4d). We further determined whether CRABP2 could modulate the expression of YAP target genes, such as CYR61 and CTGF. The results showed that the mRNA expression of CTGF, CYR61 was increased in CRABP2 deficient cells (Additional file 1: Figure S4c).

Previous studies indicate that Lats1 can prevent cells from developing EMT and preventing cancer cells from metastasis. Our study explored whether Lats1 is involved in the CRABP2-regulated EMT. Results showed that overexpression of Lats1 in T47D and MCF7 cells reduced CRABP2-induced E-cadherin and ZO-1 reduction and increase in Vimentin (Fig. 4e). Additionally, overexpression of Lats1 reversed the knocking down CRABP2 expression results promoting tumor cells invasion and metastasis by monolayer wound healing and transwell assays (Fig. 4f-h, Additional file 1: Figure S4d-f). Jointly, these data show that the regulation of CRABP2 on the EMT, metastasis, and invasion depends on Lats1 in ER^+^ mammary cancer cells.

### Overexpression of CRABP2 promotes EMT, metastasis and invasion of ER^−^ breast cancer cells by inactivating Hippo pathway

**Fig. 5:**
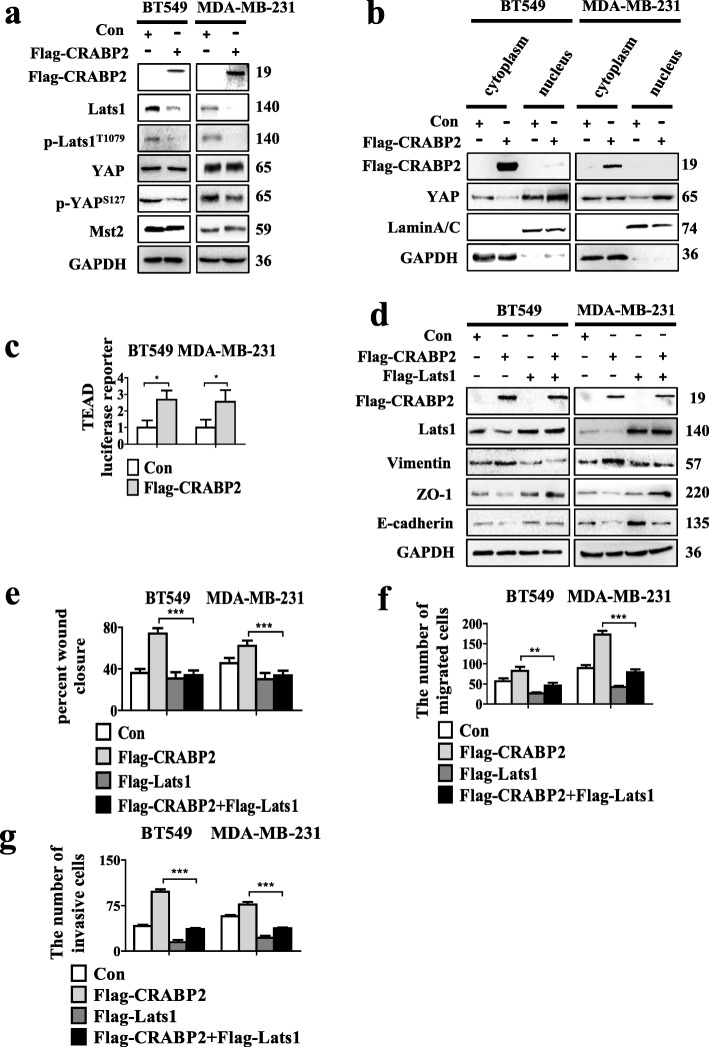
Overexpression of CRABP2 promotes EMT, metastasis and invasion of ER^−^ breast cancer by inactivating Hippo pathway. **a** Analysis of the YAP, p-YAP^s127^, Lats1, p-Lats1^T1079^ and Mst2 expression in BT549 and MDA-MB-231 cells stably expressing CRABP2 (Flag-CRABP2) and vector (Con) by Western blotting. **b** The express of YAP was analyzed in BT549 and MDA-MB-231 cells stably expressing CRABP2 (Flag-CRABP2) and vector (Con) by subcellular fractionation. Western blotting of GAPDH and LaminA/C were used as controls for cytoplasmic and nuclear fractional purity, respectively. **c** TEAD luciferase activity was analyzed in BT549 and MDA-MB-231 cells stably expressing CRABP2 (Flag-CRABP2) and vector (Con) were examined . **d** Analysis of CRABP2, Lats1, E-cadherin, ZO-1 and Vimentin expression in BT549 and MDA-MB-231 cells stably expressing CRABP2 (Flag-CRABP2) and vector (Con), with or without Lats1 (Flag-Lats1) by Western blotting. (**e**) Wound-healing, (**f**) migration and (**g**) invasion experiments were conducted in BT549 and MDA-MB-231 cells stably expressing CRABP2 (Flag-CRABP2) and vector (Con), with or without Lats1 (Flag-Lats1). The percent of wound closure and migratory and invasive cells numbers were counted. *P*-values were calculated by the Student’s *t*-test. Data are the mean ± S.D. of three independent experiments performed in triplicate. *, *P* < 0.05; **, *P* < 0.01; ***, *P* < 0.001

Likewise, it was found that knockdown of Lats1 in ER^−^ breast cancer cells did not modify the mRNA and protein expression of CRABP2 (Additional file 1: Figure S5a -b). But, overexpression of CRABP2 in ER^−^ mammary cancer cells reduced the protein levels of p-Lats1^T1079^, Lats1, p-YAP^S127^ and retained the protein expression of YAP and Mst2 (Fig. 5a). Cellular fractionation showed that overexpression CRABP2 in BT549 and MDA-MB-231 cells reduced the levels of YAP in the cytoplasm but increased the levels of YAP in nuclear when compared with controls (Fig. 5b). Then we checked the effects of CRABP2 overexpression on TEAD luciferase activity in ER^−^ breast cancer cells. We observed that CRABP2 overexpression increased TEAD luciferase activity (Fig. 5c). We further explored the expression of YAP target genes. The results showed that the mRNA expression of CTGF, CYR61 was increased in CRABP2 overexpressed cells (Additional file 1: Figure S5c). Western blotting results showed that overexpression of Lats1 in BT549 and MDA-MB-231 cells reduced overexpressed CRABP2-induced E-cadherin, ZO-1 reduction and increase in Vimentin (Fig. 5d). The monolayer wound healing and transwell assays also confirmed that overexpression of Lats1 reversed the overexpression of CRABP2 results promoting tumor cells invasion and metastasis (Fig. 5e-g, Additional file 1: Figure S5d-f). In conclusion, these data indicate that the regulation of CRABP2 on the EMT, metastasis, and invasion depends on Lats1 in ER^−^ mammary cancer cells.

### CRABP2 interacts Lats1 and regulates the degradation of Lats1 in breast cancer cells

**Fig. 6:**
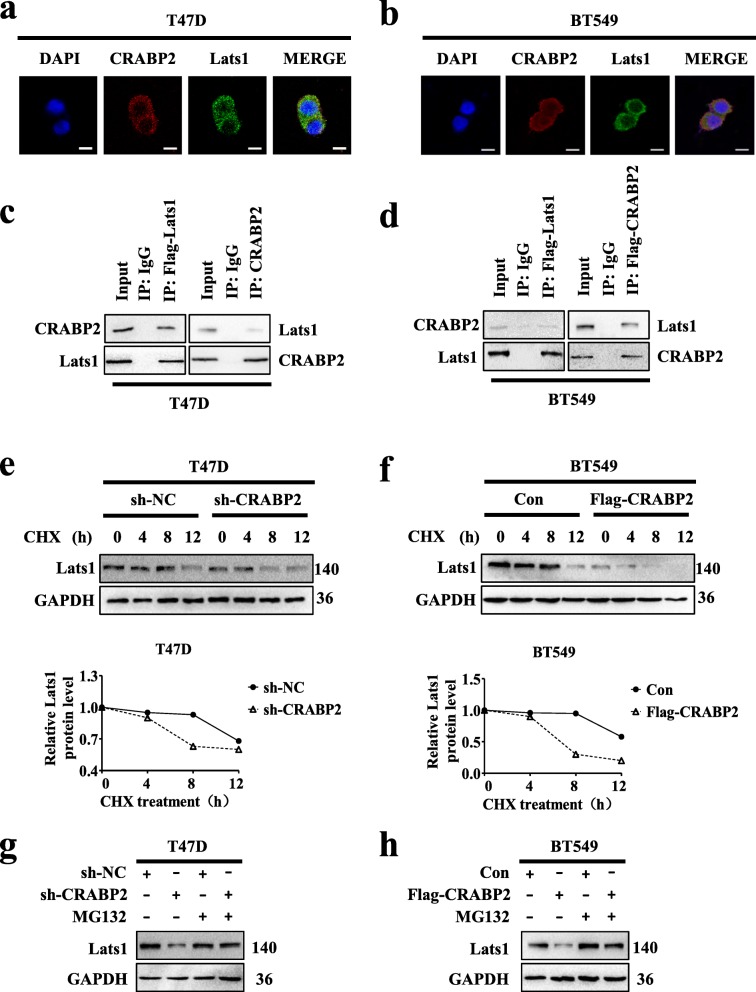
CRABP2 interacts Lats1 and regulates the degradation of Lats1 in breast cancer cells. **a-b** Subcellular localization of CRABP2 and Lats1 in T47D (**a**) and BT549 (**b**) cells by immunofluorescence. Scalebar, 10 μm. **c** In the left, extracts of T47D cell were coimmunoprecipitated (IP) with anti-Flag antibody or IgG and analyzed by anti-CRABP2 antibody with Western blotting. In the right, extracts of T47D cell were coimmunoprecipitated (IP) with anti-CRABP2 antibody or IgG and analyzed by anti-Lats1 antibody with Western blotting. **d** Detection in BT549 cells by (**c**) method. **e-f** Cycloheximide (100 μg/ml) contact T47D (**e**) and BT549 (**f**) cells with gradient time. The protein expression was examined by Western blotting. Quantification of Lats1 protein levels was determined using Image J software normalized to GAPDH (bottom panel). **g-h** MG132 (5 μM) contact T47D (**g**) and BT549 (**h**) cells with 24 h. The protein expression was examined by Western blotting. Data are the mean ± S.D. of three independent experiments performed in triplicate

RT-qPCR technique was used to examine the particular relation between CRABP2 and Lats1 in mammary cancer cells. Here we investigated the mRNA expression of Lats1 when knocking down CRABP2 in ER^+^ and ER^−^ mammary cancer cells. There was no obvious change in the mRNA expression level of Lats1 (Additional file 1: Figure S6a-b). Then, we hypothesize if CRABP2 and Lats1 are combined. Firstly, immunofluorescence experiments were carried out to test the subcellular localization of both CRABP2 and Lats1 in T47D and BT549 cells. Results showed that CRABP2 (red) and Lats1 (green) formed an overlapping staining signal (yellow) in the cytoplasm (Fig. 6a-b). Further, to prove CRABP2 as a binding partner of Lats1, we did a series of co-immunoprecipitation (co-IP) assays. The results showed that endogenous CRABP2 interacted with Lats1 in T47D (Fig. 6c), and exogenous CRABP2 interacted with Lats1 in BT549 cells (Fig. 6d) respectively. These data confirm that CRABP2 is a physiologically relevant interacting partner of Lats1. Thus, these results indicate that CRABP2 interacts with Lats1 in breast cancer cells.

From the learning that CRABP2 regulated protein expression level of Lats1, we investigated the half-lives of Lats1 under the situation of silencing or overexpressing of CRABP2 in T47D and BT549 cells. The results showed that knockdown of CRABP2 in T47D cells and overexpression of CRABP2 in BT549 cells reduced the half-life of Lats1 representing CRABP2 regulated the stability of the Lats1 (Fig. 6e-f). In general, protease degradation system is the most common protein degradation pathway and Lats1 can be degraded by ubiquitination. We hypothesize if CRABP2 affects the degradation of Lats1 by ubiquitin-dependent proteasomal degradation. As shown in Fig. 6g-h, treatment with MG132 markedly inhibited the decrease in the Lats1 protein levels in T47D cells with knocking down CRABP2 and BT549 cells with overexpressing CRABP2. Therefore, this data confirm that CRABP2 regulates the degradation of Lats1 by ubiquitin-dependent proteasomal degradation system in breast cancer cells.

### CRABP2 mediates ubiquitination of Lats1 in breast cancer cells dependent on ER status

**Fig. 7:**
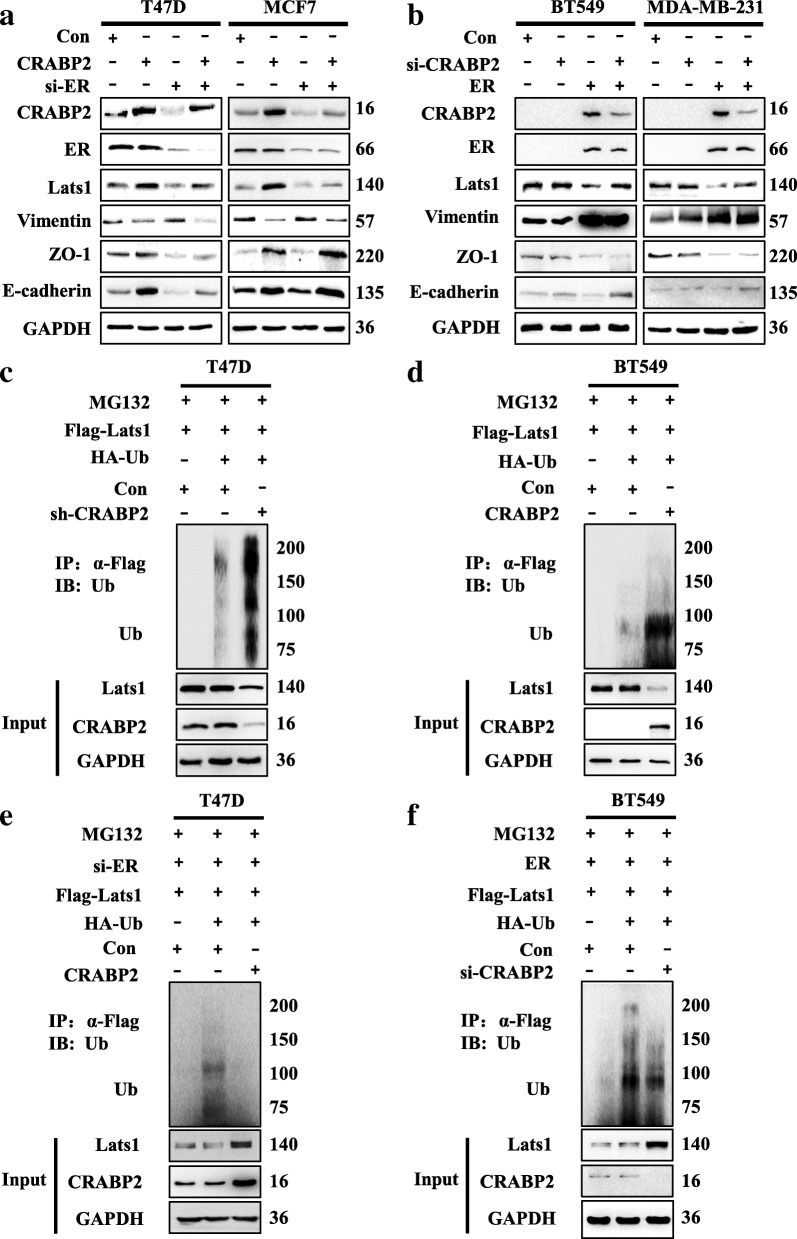
CRABP2 mediates ubiquitination of Lats1 in breast cancer cells dependent on ER status. **a** Expression of CRABP2, ER, Lats1, E-cadherin, ZO-1 and Vimentin protein was examined in T47D and MCF7 cells expressing CRABP2 (CRABP2) and vector (Con), with or without ER (si-ER) by Western blotting. **b** Expression of CRABP2, ER, Lats1, E-cadherin, ZO-1 and Vimentin protein was examined in BT549 and MDA-MB-231 cells expressing CRABP2 siRNA (si-CRABP2) and siRNA (Con), with or without ER (ER) by Western blotting. **c** The ubiquitination of Lats1 was examined in ER^+^ breast cancer cells (T47D cells) stably expressing CRABP2 shRNA (sh-CRABP2) and shRNA (sh-NC). Ubiquitin-HA and Flag-Lats1 were transfected into sh-NC and sh-CRABP2 T47D cells. **d** The ubiquitination of Lats1 was examined in ER^−^ breast cancer cells (BT549 cells) expressing CRABP2. Ubiquitin-HA, CRABP2 and Flag-Lats1 were transfected into BT549 cells. **e** The ubiquitination of Lats1 was examined when ER was knocked down and CRABP2 was overexpressed in T47D cells at the same time. Various combinations of si-ER, CRABP2 and Flag-Lats1 and ubiquitin-HA were transfected into T47D cells. **f** The ubiquitination of Lats1 was examined when ER was overexpressed and CRABP2 was knocked down in BT549 cells at the same time. Various combinations of ER, si-CRABP2 and Flag-Lats1 and ubiquitin-HA were transfected into BT549 cells. Ubiquitination Lats1 was tested by IP of Lats1 with anti-Flag antibody, and analyzed by anti-HA antibody with Western blotting

In this study, we wanted to find the relationship between CRABP2, ER and the invasion and metastasis of breast cancer. We did this by first knocking down the ER and overexpressing CRABP2 in ER^+^ cells. The results showed that E-cadherin, ZO-1 expression increased and Vimentin expression decreased when compared with the single knockdown of ER (Fig. 7a). This confirmed that knockdown of ER in ER^+^ breast cancer cells can reverse CRABP2 inhibition to the EMT of breast cancer cells. Then, we simultaneously overexpressed ER and knocked down CRABP2 in ER^−^ mammary cancer cell lines, and found that E-cadherin expression increased and ZO-1 and Vimentin expression changed a little when compared with the overexpressed ER group (Fig. 7b). This confirms that overexpression of ER in ER^−^ breast cancer cells could reverse the inhibition of knocking down CRABP2 to breast cancer cells EMT. Therefore, these findings confirm the involvement of ER in the regulation of CRABP2 on mammary cancer cells EMT.

From the previous study, it is known that knockdown of CRABP2 in ER^+^ mammary cancer cells and overexpression of CRABP2 in ER^−^ mammary cancer cells promote the degradation of Lats1. Here we hypothesize that the distinct responses also will relate to the ER. The Western blotting test was used to assess the Lats1 expression levels and to confirm the influence of ER in the regulation of CRABP2 to Lats1. The results showed that overexpression of CRABP2 increased the expression of Lats1 when ER was knocked down in ER^+^ breast cancer cells (Fig. 7a). Likewise, knockdown of CRABP2 increased the expression of Lats1 when ER was overexpressed in ER^−^ breast cancer cells (Fig. 7b).

Then we performed in vivo ubiquitination assays and the results showed that the knockdown of CRABP2 in T47D cells and the overexpression of CRABP2 in BT549 cells increased the ubiquitination of Lats1 (Fig. 7c-d). However, overexpression of CRABP2 reduced the ubiquitination of Lats1 when ER was knocked down in T47D cells (Fig. 7e). Also, knockdown of CRABP2 reduced the ubiquitination of Lats1 when ER was overexpressed in BT549 cells (Fig. 7f). Thus, from these results, we conclude that that CRABP2 mediates ubiquitination of Lats1 in mammary cancer cells relying on ER status.

## Discussion

CRABP2 regulates several biologic functions in tumors. However, how CRABP2 is associated with invasion and metastasis in breast cancer remain unclear. One report stated that messenger ribonucleic (mRNA) expression of CRABP2 in breast cancer is associated to the status of ER, progesterone receptor (PR), and human epidermal growth factor receptor 2 (Her2) [7]. Our study showed that the protein expression of CRABP2 in breast cancer is related to ER. The result also exhibited the following: epigenetic silencing of CRABP2 protein tremendously promoted tumors demonstrating a lower level of resistance ER^+^ breast cancer invasion and metastasis; Ectopic expression of CRABP2 also promoted tumors demonstrating a lower level of resistance ER^−^ breast cancer metastasis and invasion. This suggested that CRABP2 plays different roles depending on the type of breast cancer. Survival analysis was carried out using Kaplan-Meier and found that the high mRNA levels of CRABP2 were markedly associated with higher and lower patient survival probability in ER^+^ and ER^−^ breast cancer respectively. This confirms the contradiction of the correlation between CRABP2 and breast cancer patient prognosis in different studies. The reason for these differences in the role of CRABP2 may be due to the complex regulation of CRABP2 activities involving ER status.

Studies conducted in the past have indicated that ER was stabilized in the absence of Lats1, and ER was targeted for ubiquitination in the presence of Lats1 [32]. However, our results show that the protein and mRNA expression of CRABP2 did not make any visible change after knocking down Lats1. These results imply that there are many participants in ER-RARα-CRABP2-Lats1 axis. Recent laboratory data indicate that metastasis of colorectal cancer related to the Hippo pathway [13]. Therefore, we hypothesized if CRABP2 has a connection with the Hippo pathway. Our results demonstrated that CRABP2 affected the protein level and not the mRNA level of Lats1 in mammary cancer cells. CRABP2 could regulate the location of Yes-associated protein (YAP). Moreover, silencing and overexpressing CRABP2 in ER^+^ and ER^−^ mammary cancer cells induces EMT and promotes mammary cancer cells invasion and metastasis. However, overexpression of Lats1 can reverse the condition. These results show that CRABP2 regulates the invasion and metastasis of breast cancer through Lats1.

CRABP1, another member of the RA-binding protein family, was also highly expressed in MCF7 cells [7]. CRABP1 can promote the development of prostate cancer and breast cancer [35]. And CRABP1 was methylated in the majority of epithelial breast cancer cell lines [36, 37]. The previous results show that CRABP1 and CRABP2 may have different effects on breast cancer. So we hypothesized whether the biological function of CRABP2 in regulating breast cancer was related to CRABP1. To explore the relationship between CRABP1 and CRABP2, we detected the protein level of CRABP1 in sh-NC-MCF7 and sh-CRABP2-MCF7 cells. Our results showed that knockdown of CRABP2 could not change the protein expression level of CRABP1 (Additional file 1: Figure S6c). That result indicated that the effect of CRABP2 on invasion and metastasis through Hippo pathway in breast cancer was independent of CRABP1.

Our findings showed how CRABP2 alters the expression of Lats1. The interaction of endogenous and exogenous CRABP2 with Lats1 can regulate Lats1 by regulating ubiquitination of Lats1 in ER^+^ and ER^−^ mammary cancer cells respectively. With this base information, we hypothesize that the ER plays a role in the regulation of CRABP2 on Lats1. The data presented in this study suggests that CRABP2 stabilize Lats1 by inhibiting ubiquitination of Lats1 in ER^+^ mammary cancer cells. Meanwhile, CRABP2 promotes the degradation of Lats1 by promoting ubiquitination of Lats1 in ER^−^ mammary cancer cells. We guessed what the mechanism might be that ER is more capable of binding to E3 ubiquitin ligase of Lats1 than CRABP2 in ER^+^ breast cancer cells. Also, CRABP2 cannot bind to E3 ubiquitin ligase to suppress ubiquitination of Lats1 to repress invasion and metastasis of ER^+^ breast cancer. In ER^−^ mammary cancer cells, the interaction of exogenous CRABP2 and Lats1 through an E3 ubiquitin ligase promotes the ubiquitination of Lats1 to promote the invasion and metastasis of ER^−^ mammary cancer. However, after knocking ER down in ER^+^ breast cancer, the reason for that CRABP2 overexpression still can stabilize Lats1 needs to be further explored. We assume that this result might have something to do with RARα. Early studies between 1999 and 2005 reported that ER drives the transcription of retinoic acid receptor alpha (RARα). In turn, RARα drives the transcription of CRABP2 except for MDA-MB-468 cells, which belongs to ER^−^ breast cancer cells [21, 22]. However, RARα is not exclusively dependent on ERα expression [38−40]. What’s more, although the MDA-MB-468 cells were ER^−^ breast cancer cells, CRABP2 was highly expressed in MDA-MB-468 cells. We examined the effect of CRABP2 knockdown on the Hippo pathway in MDA-MB-468 cells. Our results showed that knockdown of CRABP2 in MDA-MB-468 cells could not affect Hippo pathway (Additional file 1:Figure S6d). Previous report show that MDA-MB-468 cells are a special case, because RARα, and not retinoic acid receptor gamma (RARγ), is most commonly involved in CRABP2 induced RAR isoforms [2]. These results supported that MDA-MB-468 cells are a special case in our study and show that CRABP2 regulated invasion and metastasis of breast cancer through Hippo pathway dependent on ER status may relate to RARα. However, the concrete mechanism is not clear.

**Fig. 8:**
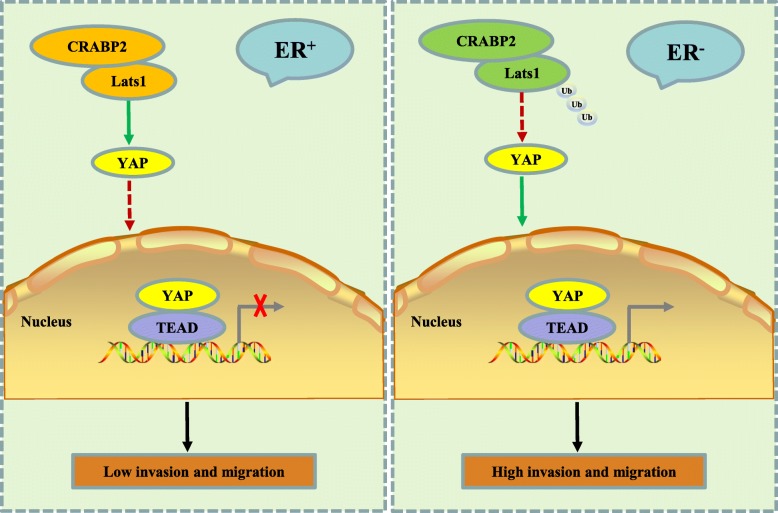
A schematic model shows how CRABP2 regulates Lats1 to affect EMT, metastasis and invasion in ER^+^ and ER^−^ breast cancer. In ER^+^ mammary cancer cells, the interaction of CRABP2 and Lats1 suppresses the ubiquitination of Lats1 to activate Hippo pathway to inhibit the invasion and metastasis of ER^+^ mammary cancer. However, in ER^−^ mammary cancer cells, the interaction of CRABP2 and Lats1 promotes the ubiquitination of Lats1 to inactivate Hippo pathway to promote the invasion and metastasis of ER^−^ mammary cancer

In conclusion, Fig. 8 represents the models of the relationship between CRABP2, Lats1, ER, invasion, and metastasis of mammary cancer. In ER^+^ mammary cancer cells, the interaction of CRABP2 and Lats1 suppresses the ubiquitination of Lats1 to activate Hippo pathway to inhibit the invasion and metastasis of ER^+^ mammary cancer. However, in ER^−^ mammary cancer cells, the interaction of CRABP2 and Lats1 promotes the ubiquitination of Lats1 to inactivate Hippo pathway to promote the invasion and metastasis of ER^−^ mammary cancer. Several evidences have been collected to indicate the behavioral characteristic in cells depends on ER status. For example, ER^+^ and ER^−^ breast cancer subtypes respond differently to hypoxic exposure [41]. The regulation of metastasis and stem cell-like activity by cyclin D1 and cyclin-dependent kinase 4/6 (CDK4/6) relies on the expression of ER [42].

CRABP2, a kind of RA binding protein and hence its expression is also affected by RA to some extent. However, how RA regulates CRABP2 in mammary cancer invasion and metastasis requires further investigation.

Indeed, treatment for breast cancer has made great progress but common treatment method does not work effectively for most of the patients resulting in recurrence. It is, therefore, crucial to understanding the occurrence and progression of breast cancer to advance the treatment method and improve the cure rate of patients. From this study, it is understood that CRABP2 regulates invasion and metastasis diversely in different cell types. Therefore, moving forward we recommend the importance of cell types among different patients to be considered both experimentally and clinically in providing treatment.

## Conclusion

In general, our study has identified the role of CRABP2 in breast cancer invasion and metastasis, which further depends on Hippo-Lats1 and ER status. This indicates that the treatment of breast cancer should be divided into different types. Our study results have laid a positive foundation for breast cancer therapy.

## Additional files

Additional file 1: **Figure S1.** **a** Knockdown of ER in ER^+^ breast cancer cells down-regulate the protein expression of CRABP2. **b** Overexpression of ER in ER^+^ breast cancer cells up-regulate the protein expression of CRABP2.** c-f** We have constructed stable knockdown and overexpressed CRABP2 cells.**Figure S2**. **a-c** Knockdown of CRABP2 promotes metastasis and invasion of ER^+^ breast cancer cells *in vitro*. **d** Ectopic expression of CRABP2 in MCF7 cells can suppress EMT and activate Hippo pathway.**Figure S3.** **a-c** Overexpression of CRABP2 promotes metastasis and invasion of ER^-^ breast cancer cells *in vitro*.**Figure S4. a-b** Knockdown of Lats1 in ER^+^ breast cancer cells could not modify the mRNA and protein expression of CRABP2. **c** The mRNA expression of CTGF, CYR61 was increased in CRABP2 deficient cells. **d-f** The regulation of CRABP2 on metastasis, and invasion depends on Lats1 in ER^+^ mammary cancer cells. **Figure S5. a-b** Knockdown of Lats1 in ER^-^ breast cancer cells could not modify the mRNA and protein expression of CRABP2. **c** The mRNA expression of CTGF, CYR61 was increased in CRABP2 overexpressed cells. **d-f** The regulation of CRABP2 on the metastasis, and invasion depends on Lats1 in ER^-^ mammary cancer cells. **Figure S6. a-b** There was no obvious change in the mRNA expression level of Lats1 when knocking down CRABP2 in ER^+^ and ER^-^ mammary cancer cells. **c** Knockdown of CRABP2 in MCF7 cells could not modify the protein expression of CRABP1. **d** Knockdown of CRABP2 in MDA-MB-468 cells could not regulate the Hippo pathway.

Additional file 2: **Table S1.** Clinicopathological associations of CRABP2 in human breast cancers. **Table S2.** The sequences of primer set for real-time PCR assays. **Table S3.** The sequences of shRNA and siRNA used in this study. (ZIP 247 kb)

## Data Availability

Not applicable.

## Abbreviations

AREG: Amphiregulin
BSA: Bull Serum Albumin
CDK4/6: Cyclin-dependent kinase 4/6
CRABP2: Cellular retinoic acid binding protein 2
CTGF: Connective tissue growth factor
EMT: Epithelial-mesenchymal transition
ERα: Estrogen receptor-α
ESCC: Esophageal squamous cell carcinoma
FABP: Fatty acid binding protein
GAPDH: Glyceraldehyde-3-phosphate dehydrogenase
Her2: Human epidermal growth factor receptor 2
HNSCC: Head and neck squamous-cell carcinoma
Lats1: Large tumor suppressor 1
NSCLC: Non-small cell lung cancer
PBS: Phosphate buffered saline
PR: Progesterone receptor
PVDF: Polyvinyl difluoride
RA: Retinoic acid
RAR: Retinoic acid receptor
RFS: Relapse-free survival
RT-qPCR: Quantitative reverse transcription polymerase chain reaction
SSR: Sex steroid receptor
TNBC: Triple Negative Breast cancer
YAP: Yes-associated protein
