# Supplementary figures and images for: CRABP2 regulates invasion and metastasis of breast cancer through hippo pathway dependent on ER status
Source: J Exp Clin Cancer Res. 2019 Aug 16;38:361. doi: 10.1186/s13046-019-1345-2 (PMC6697986; doi:10.1186/s13046-019-1345-2)

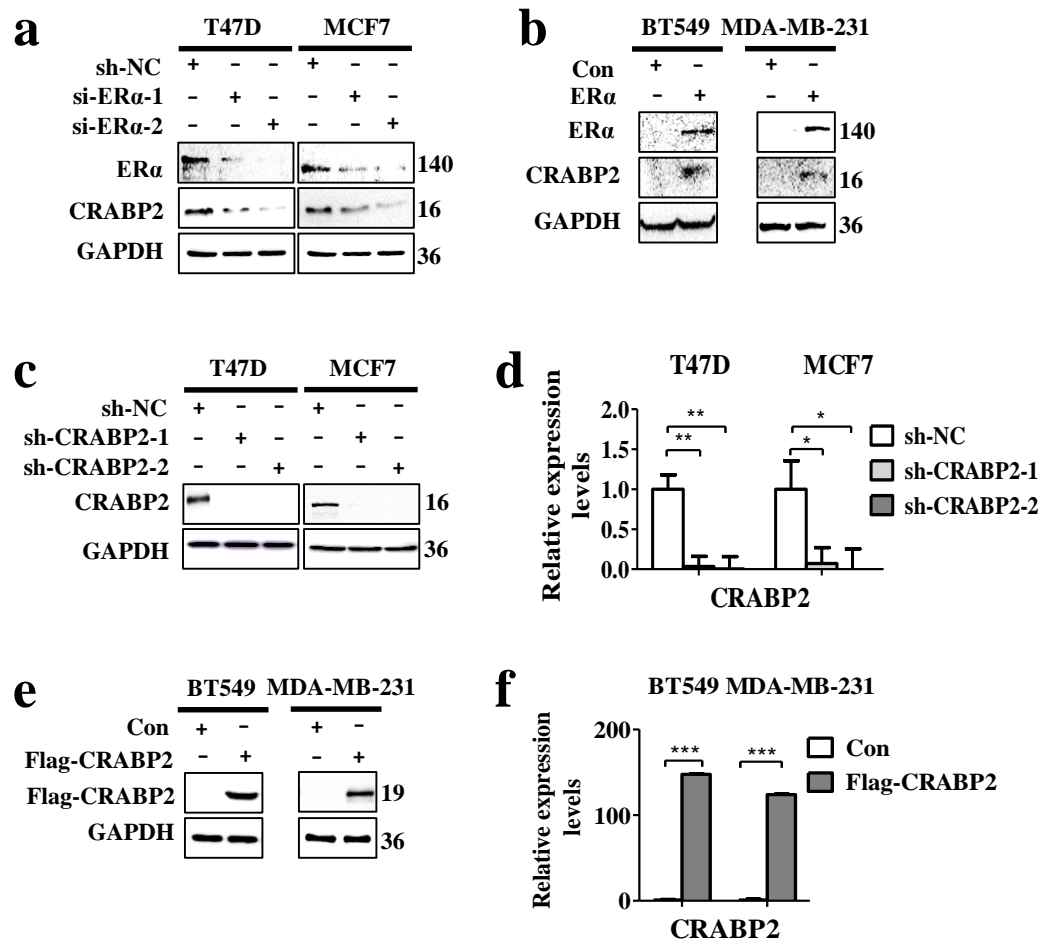

Additional file 1 : Figure S1

Supplement: Supplementary file 1 — Figure S1. a Knockdown of ER in ER+ breast cancer cells down-regulate the protein expression of CRABP2. b Overexpression of ER in ER+ breast cancer cells up-regulate the protein expression of CRABP2. c-f We have constructed stable knockdown and overexpressed CRABP2 cells.Figure S2. a-c Knockdown of CRABP2 promotes metastasis and invasion of ER+ breast cancer cells in vitro. d Ectopic expression of CRABP2 in MCF7 cells can suppress EMT and activate Hippo pathway.Figure S3. a-c Overexpression of CRABP2 promotes metastasis and invasion of ER- breast cancer cells in vitro.Figure S4. a-b Knockdown of Lats1 in ER+ breast cancer cells could not modify the mRNA and protein expression of CRABP2. c The mRNA expression of CTGF, CYR61 was increased in CRABP2 deficient cells. d-f The regulation of CRABP2 on metastasis, and invasion depends on Lats1 in ER+ mammary cancer cells. Figure S5. a-b Knockdown of Lats1 in ER- breast cancer cells could not modify the mRNA and protein expression of CRABP2. c The mRNA expression of CTGF, CYR61 was increased in CRABP2 overexpressed cells. d-f The regulation of CRABP2 on the metastasis, and invasion depends on Lats1 in ER- mammary cancer cells. Figure S6. a-b There was no obvious change in the mRNA expression level of Lats1 when knocking down CRABP2 in ER+ and ER- mammary cancer cells. c Knockdown of CRABP2 in MCF7 cells could not modify the protein expression of CRABP1. d Knockdown of CRABP2 in MDA-MB-468 cells could not regulate the Hippo pathway. [file 13046_2019_1345_MOESM1_ESM.zip › FigureS1.pdf]

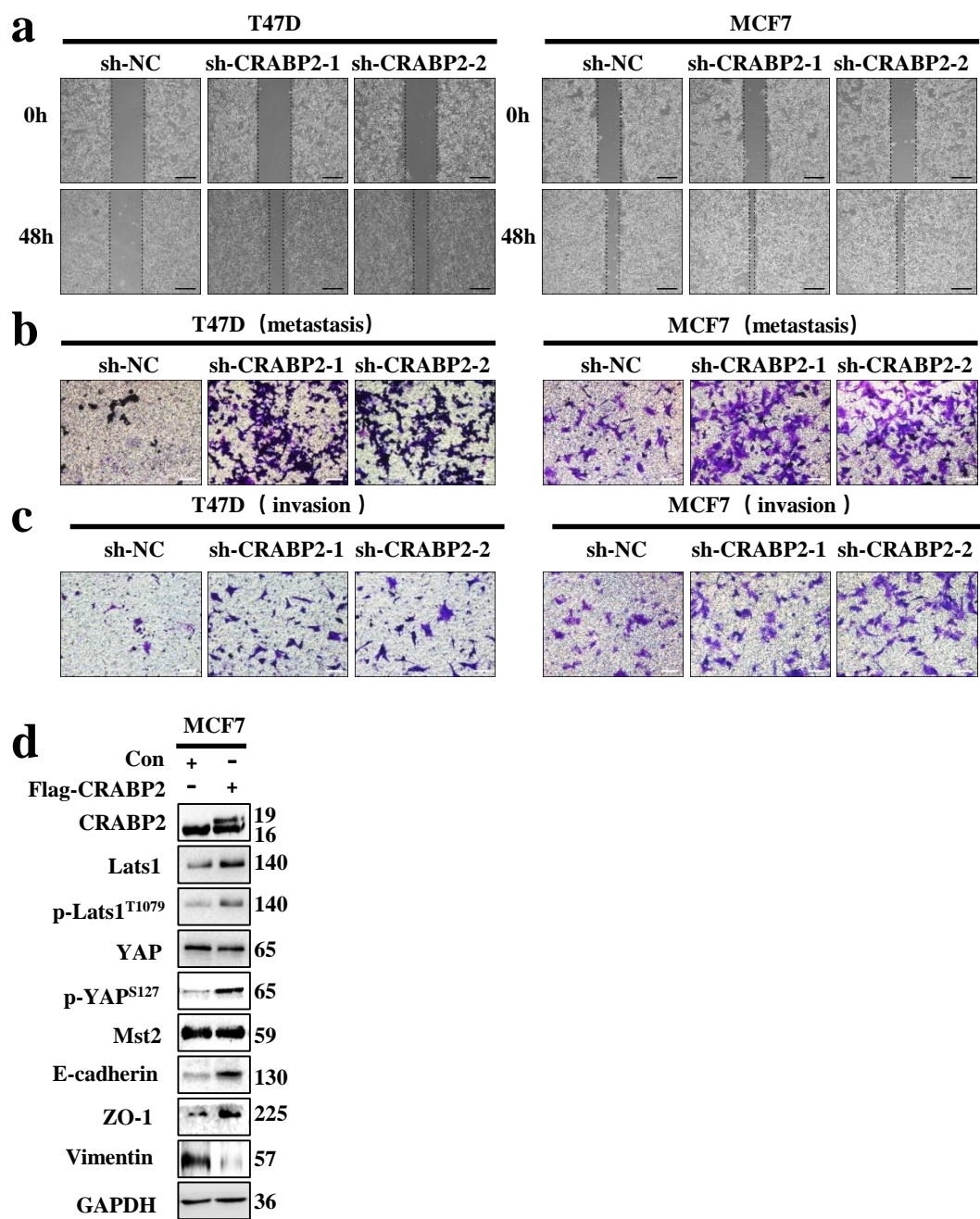

Additional file 1: Figure S2

Supplement: Supplementary file 1 — Figure S1. a Knockdown of ER in ER+ breast cancer cells down-regulate the protein expression of CRABP2. b Overexpression of ER in ER+ breast cancer cells up-regulate the protein expression of CRABP2. c-f We have constructed stable knockdown and overexpressed CRABP2 cells.Figure S2. a-c Knockdown of CRABP2 promotes metastasis and invasion of ER+ breast cancer cells in vitro. d Ectopic expression of CRABP2 in MCF7 cells can suppress EMT and activate Hippo pathway.Figure S3. a-c Overexpression of CRABP2 promotes metastasis and invasion of ER- breast cancer cells in vitro.Figure S4. a-b Knockdown of Lats1 in ER+ breast cancer cells could not modify the mRNA and protein expression of CRABP2. c The mRNA expression of CTGF, CYR61 was increased in CRABP2 deficient cells. d-f The regulation of CRABP2 on metastasis, and invasion depends on Lats1 in ER+ mammary cancer cells. Figure S5. a-b Knockdown of Lats1 in ER- breast cancer cells could not modify the mRNA and protein expression of CRABP2. c The mRNA expression of CTGF, CYR61 was increased in CRABP2 overexpressed cells. d-f The regulation of CRABP2 on the metastasis, and invasion depends on Lats1 in ER- mammary cancer cells. Figure S6. a-b There was no obvious change in the mRNA expression level of Lats1 when knocking down CRABP2 in ER+ and ER- mammary cancer cells. c Knockdown of CRABP2 in MCF7 cells could not modify the protein expression of CRABP1. d Knockdown of CRABP2 in MDA-MB-468 cells could not regulate the Hippo pathway. [file 13046_2019_1345_MOESM1_ESM.zip › FigureS2.pdf]

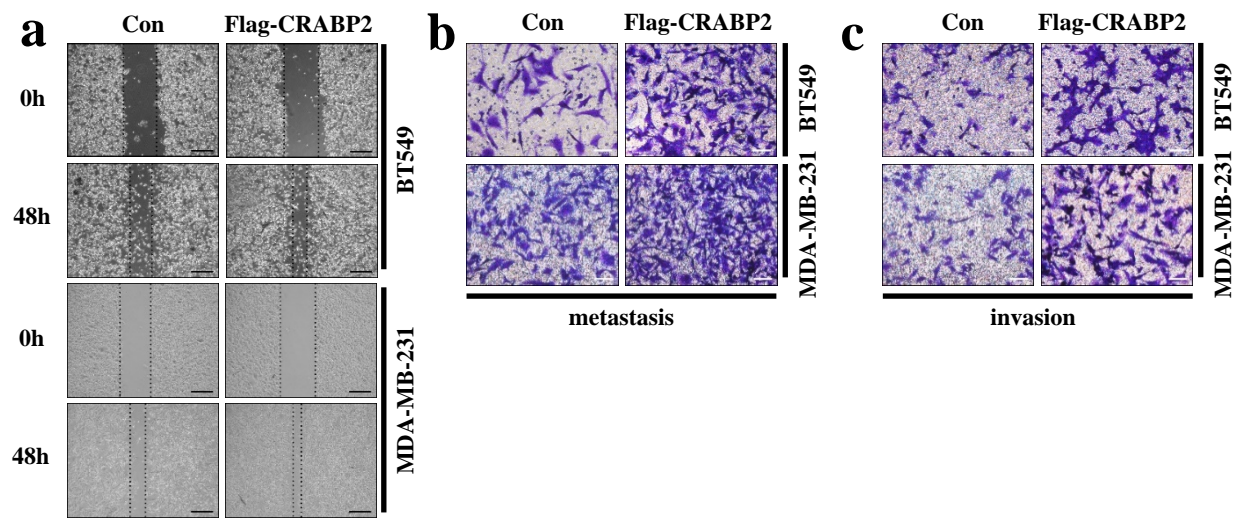

Additional file 1 : Figure S3

Supplement: Supplementary file 1 — Figure S1. a Knockdown of ER in ER+ breast cancer cells down-regulate the protein expression of CRABP2. b Overexpression of ER in ER+ breast cancer cells up-regulate the protein expression of CRABP2. c-f We have constructed stable knockdown and overexpressed CRABP2 cells.Figure S2. a-c Knockdown of CRABP2 promotes metastasis and invasion of ER+ breast cancer cells in vitro. d Ectopic expression of CRABP2 in MCF7 cells can suppress EMT and activate Hippo pathway.Figure S3. a-c Overexpression of CRABP2 promotes metastasis and invasion of ER- breast cancer cells in vitro.Figure S4. a-b Knockdown of Lats1 in ER+ breast cancer cells could not modify the mRNA and protein expression of CRABP2. c The mRNA expression of CTGF, CYR61 was increased in CRABP2 deficient cells. d-f The regulation of CRABP2 on metastasis, and invasion depends on Lats1 in ER+ mammary cancer cells. Figure S5. a-b Knockdown of Lats1 in ER- breast cancer cells could not modify the mRNA and protein expression of CRABP2. c The mRNA expression of CTGF, CYR61 was increased in CRABP2 overexpressed cells. d-f The regulation of CRABP2 on the metastasis, and invasion depends on Lats1 in ER- mammary cancer cells. Figure S6. a-b There was no obvious change in the mRNA expression level of Lats1 when knocking down CRABP2 in ER+ and ER- mammary cancer cells. c Knockdown of CRABP2 in MCF7 cells could not modify the protein expression of CRABP1. d Knockdown of CRABP2 in MDA-MB-468 cells could not regulate the Hippo pathway. [file 13046_2019_1345_MOESM1_ESM.zip › FigureS3.pdf]

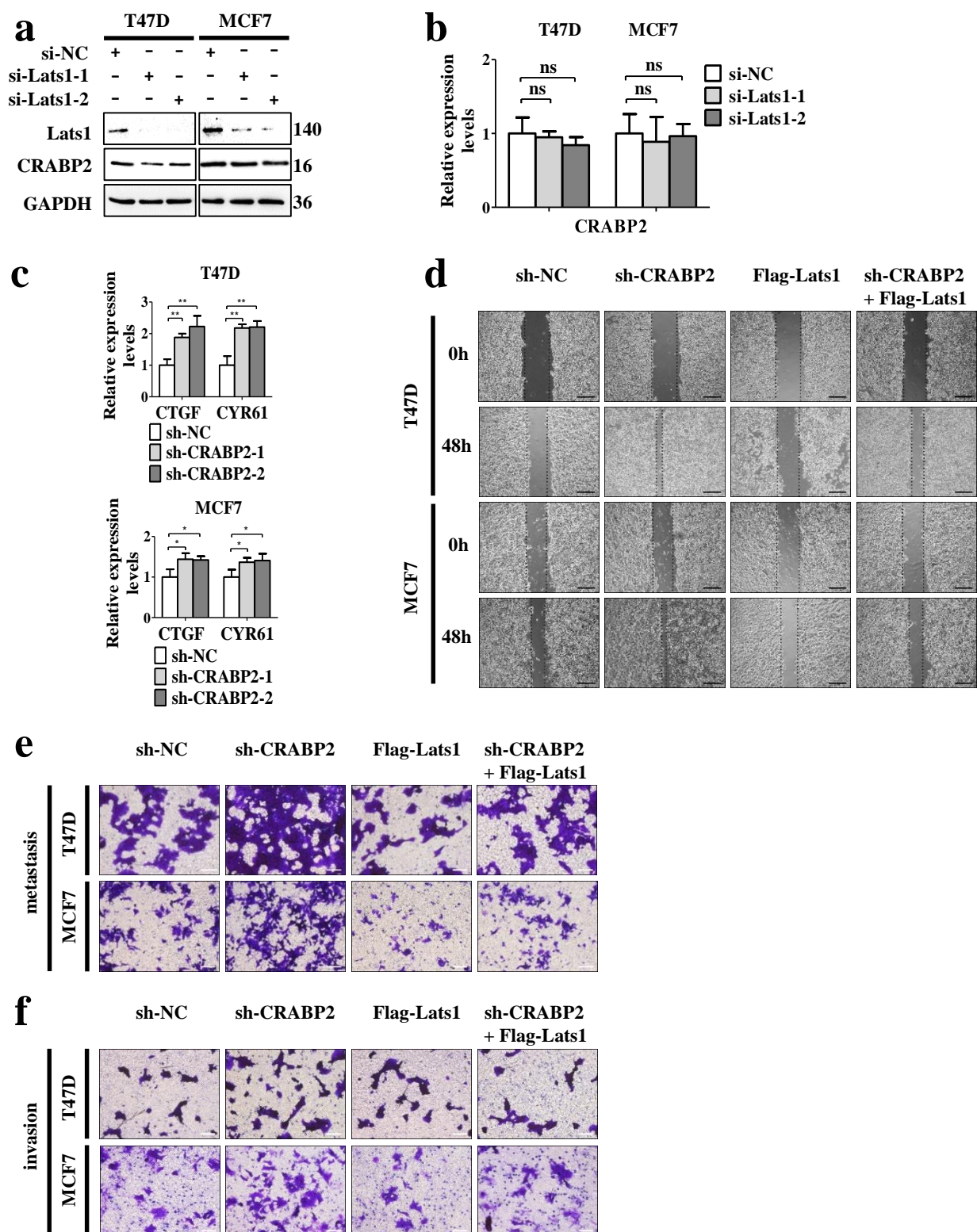

Additional file 1: Figure S4

Supplement: Supplementary file 1 — Figure S1. a Knockdown of ER in ER+ breast cancer cells down-regulate the protein expression of CRABP2. b Overexpression of ER in ER+ breast cancer cells up-regulate the protein expression of CRABP2. c-f We have constructed stable knockdown and overexpressed CRABP2 cells.Figure S2. a-c Knockdown of CRABP2 promotes metastasis and invasion of ER+ breast cancer cells in vitro. d Ectopic expression of CRABP2 in MCF7 cells can suppress EMT and activate Hippo pathway.Figure S3. a-c Overexpression of CRABP2 promotes metastasis and invasion of ER- breast cancer cells in vitro.Figure S4. a-b Knockdown of Lats1 in ER+ breast cancer cells could not modify the mRNA and protein expression of CRABP2. c The mRNA expression of CTGF, CYR61 was increased in CRABP2 deficient cells. d-f The regulation of CRABP2 on metastasis, and invasion depends on Lats1 in ER+ mammary cancer cells. Figure S5. a-b Knockdown of Lats1 in ER- breast cancer cells could not modify the mRNA and protein expression of CRABP2. c The mRNA expression of CTGF, CYR61 was increased in CRABP2 overexpressed cells. d-f The regulation of CRABP2 on the metastasis, and invasion depends on Lats1 in ER- mammary cancer cells. Figure S6. a-b There was no obvious change in the mRNA expression level of Lats1 when knocking down CRABP2 in ER+ and ER- mammary cancer cells. c Knockdown of CRABP2 in MCF7 cells could not modify the protein expression of CRABP1. d Knockdown of CRABP2 in MDA-MB-468 cells could not regulate the Hippo pathway. [file 13046_2019_1345_MOESM1_ESM.zip › FigureS4.pdf]

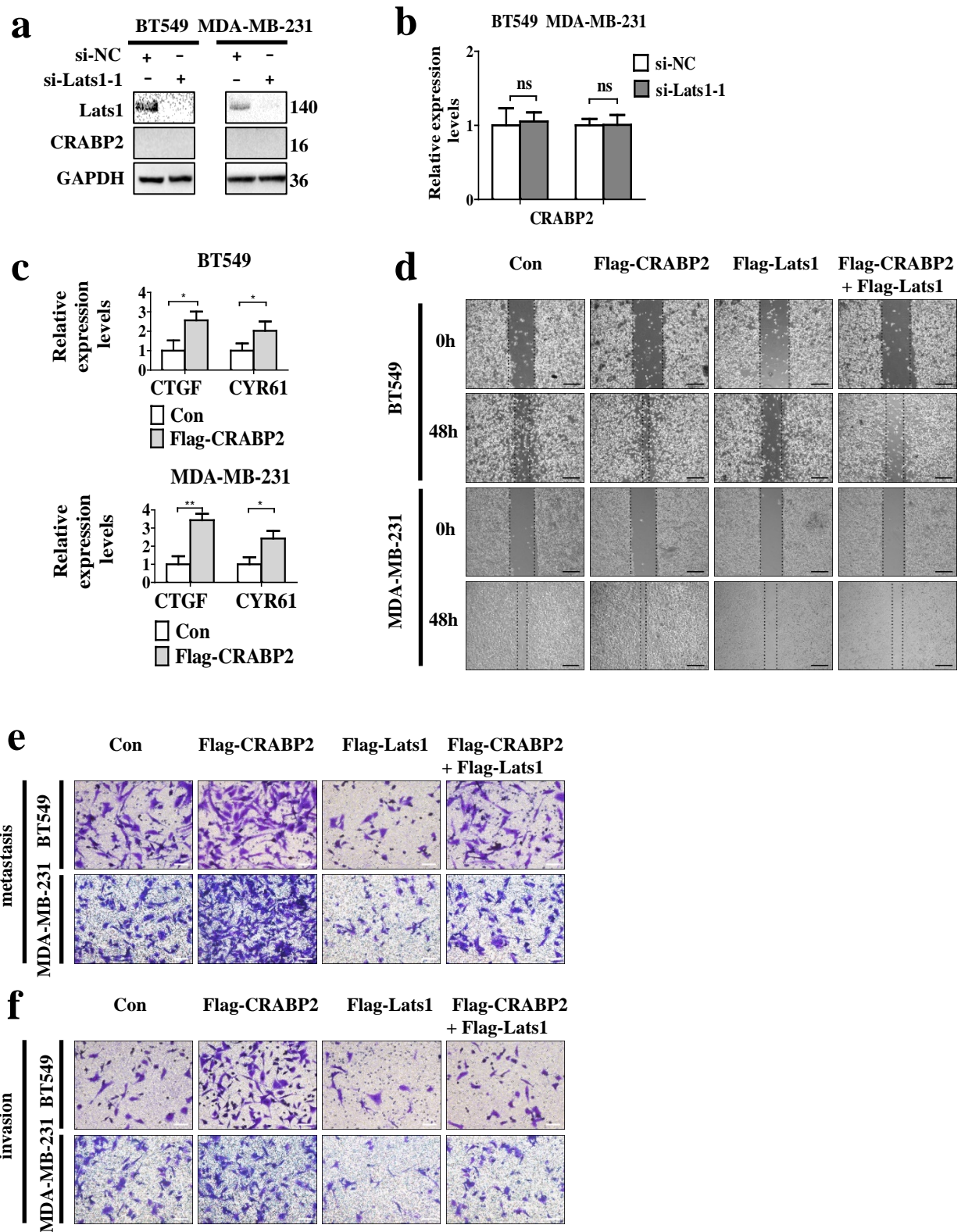

Additional file 1 : Figure S5

Supplement: Supplementary file 1 — Figure S1. a Knockdown of ER in ER+ breast cancer cells down-regulate the protein expression of CRABP2. b Overexpression of ER in ER+ breast cancer cells up-regulate the protein expression of CRABP2. c-f We have constructed stable knockdown and overexpressed CRABP2 cells.Figure S2. a-c Knockdown of CRABP2 promotes metastasis and invasion of ER+ breast cancer cells in vitro. d Ectopic expression of CRABP2 in MCF7 cells can suppress EMT and activate Hippo pathway.Figure S3. a-c Overexpression of CRABP2 promotes metastasis and invasion of ER- breast cancer cells in vitro.Figure S4. a-b Knockdown of Lats1 in ER+ breast cancer cells could not modify the mRNA and protein expression of CRABP2. c The mRNA expression of CTGF, CYR61 was increased in CRABP2 deficient cells. d-f The regulation of CRABP2 on metastasis, and invasion depends on Lats1 in ER+ mammary cancer cells. Figure S5. a-b Knockdown of Lats1 in ER- breast cancer cells could not modify the mRNA and protein expression of CRABP2. c The mRNA expression of CTGF, CYR61 was increased in CRABP2 overexpressed cells. d-f The regulation of CRABP2 on the metastasis, and invasion depends on Lats1 in ER- mammary cancer cells. Figure S6. a-b There was no obvious change in the mRNA expression level of Lats1 when knocking down CRABP2 in ER+ and ER- mammary cancer cells. c Knockdown of CRABP2 in MCF7 cells could not modify the protein expression of CRABP1. d Knockdown of CRABP2 in MDA-MB-468 cells could not regulate the Hippo pathway. [file 13046_2019_1345_MOESM1_ESM.zip › FigureS5.pdf]

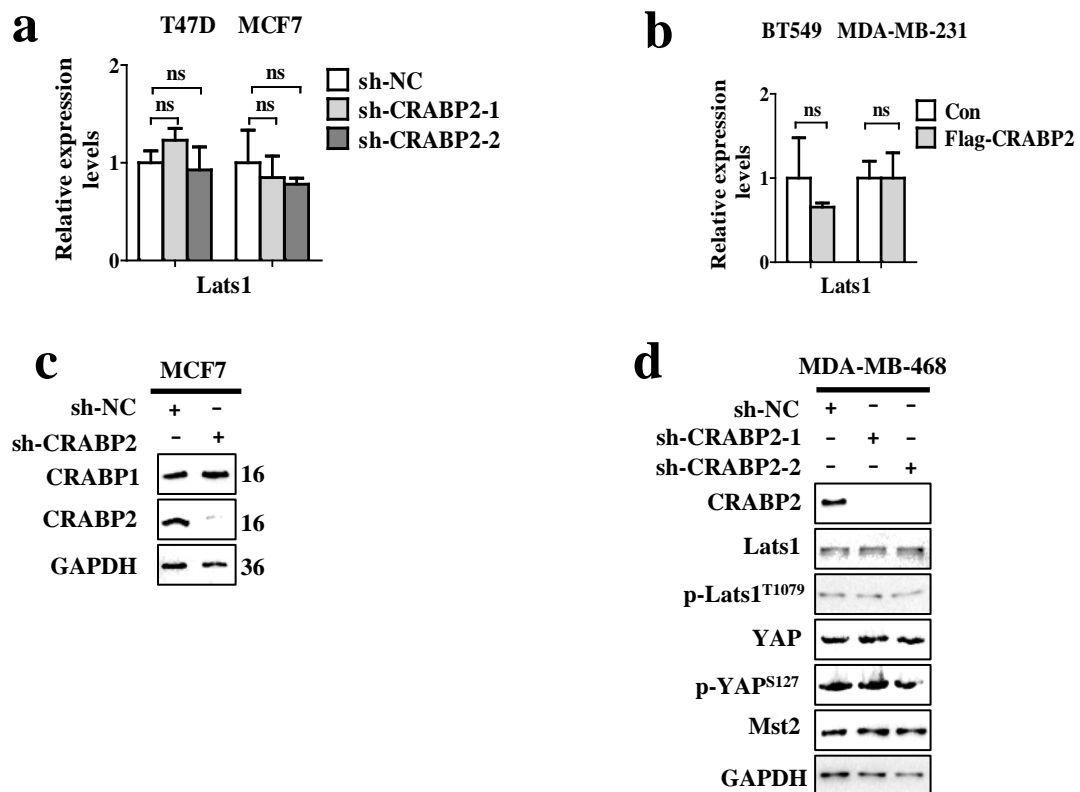

Additional file 1 : Figure S6

Supplement: Supplementary file 1 — Figure S1. a Knockdown of ER in ER+ breast cancer cells down-regulate the protein expression of CRABP2. b Overexpression of ER in ER+ breast cancer cells up-regulate the protein expression of CRABP2. c-f We have constructed stable knockdown and overexpressed CRABP2 cells.Figure S2. a-c Knockdown of CRABP2 promotes metastasis and invasion of ER+ breast cancer cells in vitro. d Ectopic expression of CRABP2 in MCF7 cells can suppress EMT and activate Hippo pathway.Figure S3. a-c Overexpression of CRABP2 promotes metastasis and invasion of ER- breast cancer cells in vitro.Figure S4. a-b Knockdown of Lats1 in ER+ breast cancer cells could not modify the mRNA and protein expression of CRABP2. c The mRNA expression of CTGF, CYR61 was increased in CRABP2 deficient cells. d-f The regulation of CRABP2 on metastasis, and invasion depends on Lats1 in ER+ mammary cancer cells. Figure S5. a-b Knockdown of Lats1 in ER- breast cancer cells could not modify the mRNA and protein expression of CRABP2. c The mRNA expression of CTGF, CYR61 was increased in CRABP2 overexpressed cells. d-f The regulation of CRABP2 on the metastasis, and invasion depends on Lats1 in ER- mammary cancer cells. Figure S6. a-b There was no obvious change in the mRNA expression level of Lats1 when knocking down CRABP2 in ER+ and ER- mammary cancer cells. c Knockdown of CRABP2 in MCF7 cells could not modify the protein expression of CRABP1. d Knockdown of CRABP2 in MDA-MB-468 cells could not regulate the Hippo pathway. [file 13046_2019_1345_MOESM1_ESM.zip › FigureS6.pdf]
