# Supplementary material for: CRABP2 regulates invasion and metastasis of breast cancer through hippo pathway dependent on ER status
Source: J Exp Clin Cancer Res. 2019 Aug 16;38:361. doi: 10.1186/s13046-019-1345-2 (PMC6697986; doi:10.1186/s13046-019-1345-2)
Supplement: Supplementary file 2 — Table S1. Clinicopathological associations of CRABP2 in human breast cancers. Table S2. The sequences of primer set for real-time PCR assays. Table S3. The sequences of shRNA and siRNA used in this study. (ZIP 247 kb) [file 13046_2019_1345_MOESM2_ESM.zip › TableS1.pdf]

Supplementary Table 1 Clinicopathological associations of CRABP2 in human breast cancers.

Clinicopathologic parameter	Total no.	CRABP2 Low(%)	CRABP2 High(%)	*p*
ER				**0.0016***
Negative	36	13（36%）	23（64%）	
Positive	61	6 （10%）	55（90%）	
PR				0.2587
Negative	50	12（24%）	38（76%）	
Positive	47	7 （15%）	40（85%）	
Her2				0.8963
Negative	37	7 （19%）	30（81%）	
Positive	60	12（20%）	48（80%）	
Triple				0.1035
No	79	13（16%）	66（84%）	
Yes	18	6 （33%）	12（67%）	
Cancer status				0.2059
Ⅱ	42	19（45%）	23（55%）	
Ⅲ	55	32（58%）	23（42%）	
Tumor size(cm)				0.7327
<2	47	20（43%）	27（57%）	
≥2	50	23（46%）	27（54%）	
Tumor site				0.7931
Left	45	17（38%）	28（62%）	
Right	52	21（40%）	31（60%）	

**P*<0.05, statistically significant

Additional file 2：Table S1
