# Supplementary material for: CRABP2 regulates invasion and metastasis of breast cancer through hippo pathway dependent on ER status
Source: J Exp Clin Cancer Res. 2019 Aug 16;38:361. doi: 10.1186/s13046-019-1345-2 (PMC6697986; doi:10.1186/s13046-019-1345-2)
Supplement: Supplementary file 2 — Table S1. Clinicopathological associations of CRABP2 in human breast cancers. Table S2. The sequences of primer set for real-time PCR assays. Table S3. The sequences of shRNA and siRNA used in this study. (ZIP 247 kb) [file 13046_2019_1345_MOESM2_ESM.zip › TableS2.pdf]

Supplementary Table 2 The sequence of primer sets for real- time PCR assay.

ID	Forward（5'-3'）	Reverse（5'-3'）
**CTGF**	5′-AGGTGTGGCTTTAGGAGCAG-3′	5′-TCTTGATGGCTGGAGAATGC-3′
**CYR61**	5′- TGGAACTGGTATCTCCACACG-3′	5′-TACACTGGCTGTCCACAAGG-3′
**CRABP2**	5′- AGTGTCCAGTGCTCCAGCCTA-3′	5′-CTGCAGCCACAGCAATCTTC-3′
**Lats1**	5′-TGTCCAGGAGCTCTGCTCTC-3′	5′-CCACACGAAGGACTTCTTTATTTGA-3′
**GAPDH**	5′-CTCCTCCACCTTTGACGCTG-3′	5′-CTCCTCCACCTTTGACGCTG-3′

Additional file 2：Table S2
