# Supplementary material for: CRABP2 regulates invasion and metastasis of breast cancer through hippo pathway dependent on ER status
Source: J Exp Clin Cancer Res. 2019 Aug 16;38:361. doi: 10.1186/s13046-019-1345-2 (PMC6697986; doi:10.1186/s13046-019-1345-2)
Supplement: Supplementary file 2 — Table S1. Clinicopathological associations of CRABP2 in human breast cancers. Table S2. The sequences of primer set for real-time PCR assays. Table S3. The sequences of shRNA and siRNA used in this study. (ZIP 247 kb) [file 13046_2019_1345_MOESM2_ESM.zip › TableS3.pdf]

Supplementary Table 3 The sequence of shRNA and siRNA.

ID	sense（5'-3'）	antisense（5'-3'）
**sh-CRABP2- homo-1**	GCACCACAGAGAUUAACUUTT	AAGUUAAUCUCUGUGGUGCTT
**sh-CRABP2- homo-2**	GGGAGAGUGAGAAUAAAAUTT	AUUUUAUUCUCACUCUCCCTT
**si- ESR1-homo-1**	GCAUUCUACAGGCCAAAUUTT	AAUUUGGCCUGUAGAAUGCTT
**si- ESR1-homo-2**	GGAGAAUGUUGAAACACAATT	UUGUGUUUCAACAUUCUCCTT
**si-Lats1- homo-1**	GAGCUGGAAAGGUUCUAAATT	UUUAGAACCUUUCCAGCUCTT
**si-Lats1- homo-2**	GCAGCGUCUACAUCGUAAATT	UUUACGAUGUAGACGCUGCTT

Additional file 2：Table S3
